# 2-Amino-4-methyl-4,5,6,7-tetrahydro-1-benzothiophene-3-carbonitrile

**DOI:** 10.1107/S160053681204010X

**Published:** 2012-09-26

**Authors:** Ashraf Y. Khan, Nikhath Fathima, Mallikarjun B. Kalashetti, Noor Shahina Begum, I. M. Khazi

**Affiliations:** a Department of Chemistry, Karnatak University, Dharwad 580 003, India; b Department of Studies in Chemistry, Bangalore University, Bangalore 560 001, Karnataka, India

## Abstract

In the title compound, C_10_H_12_N_2_S, the thio­phene ring is essentially planar (r.m.s. deviation = 0.0290 Å). The two C atoms of the cyclo­hexene ring (at positions 6 and 7) are disordered over two sets of sites in a 0.810 (5):0.190 (5) ratio. The cyclo­hexene rings in both the major and minor occupancy conformers adopt a half-chair conformation. In the crystal, there are two types of N—H⋯N inter­action. One of these results in centrosymmetric head-to-head dimers corresponding to an *R*_2_^2^(12) graph-set motif and the other forms a 20-membered macrocyclic ring involving six mol­ecules.

## Related literature

For biological activities of benzothio­phenes, see: Shetty *et al.* (2009 ▶). For the crystal structure of a closely related compound, see: Ziaulla *et al.* (2011 ▶). For graph-set notation of hydrogen bonds, see: Bernstein *et al.* (1995 ▶).
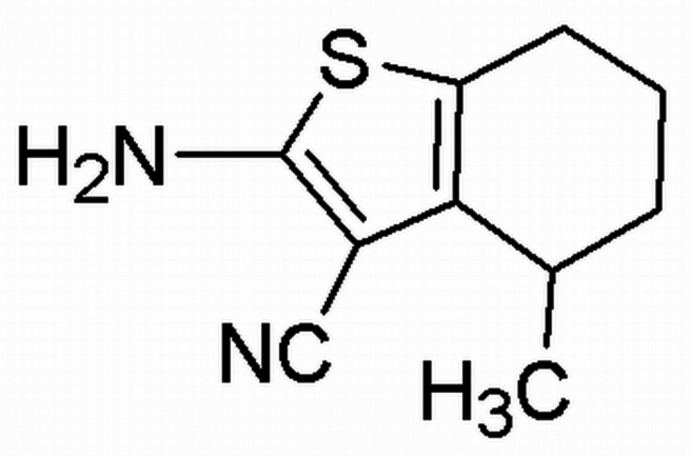


## Experimental

### 

#### Crystal data

C_10_H_12_N_2_S
*M* *_r_* = 192.28
Monoclinic, 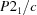

*a* = 9.6771 (2) Å
*b* = 7.6364 (2) Å
*c* = 13.8156 (3) Å
β = 100.221 (2)°
*V* = 1004.75 (4) Å^3^
*Z* = 4
Mo *K*α radiation
μ = 0.28 mm^−1^
*T* = 296 K
0.18 × 0.16 × 0.16 mm

#### Data collection

Bruker SMART APEX CCD detector diffractometer
Absorption correction: multi-scan (*SADABS*; Bruker, 1998 ▶) *T*_min_ = 0.952, *T*_max_ = 0.957
8861 measured reflections
2195 independent reflections
1812 reflections with *I* > 2σ(*I*)
*R*_int_ = 0.024

#### Refinement

*R*[*F* ^2^ > 2σ(*F* ^2^)] = 0.039
*wR*(*F* ^2^) = 0.118
*S* = 1.06
2195 reflections
125 parameters
H-atom parameters constrained
Δρ_max_ = 0.16 e Å^−3^
Δρ_min_ = −0.25 e Å^−3^

### 

Data collection: *SMART* (Bruker, 1998 ▶); cell refinement: *SMART*; data reduction: *SAINT-Plus* (Bruker, 1998 ▶); program(s) used to solve structure: *SHELXS97* (Sheldrick, 2008 ▶); program(s) used to refine structure: *SHELXL97* (Sheldrick, 2008 ▶); molecular graphics: *ORTEP-3* (Farrugia, 1997 ▶) and *CAMERON* (Watkin *et al.*, 1996 ▶); software used to prepare material for publication: *WinGX* (Farrugia, 1999 ▶).

## Supplementary Material

Crystal structure: contains datablock(s) global, I. DOI: 10.1107/S160053681204010X/pv2585sup1.cif

Structure factors: contains datablock(s) I. DOI: 10.1107/S160053681204010X/pv2585Isup2.hkl

Supplementary material file. DOI: 10.1107/S160053681204010X/pv2585Isup3.cml

Additional supplementary materials:  crystallographic information; 3D view; checkCIF report

## Figures and Tables

**Table 1:** Hydrogen-bond geometry (Å, °)

*D*—H⋯*A*	*D*—H	H⋯*A*	*D*⋯*A*	*D*—H⋯*A*
N1—H1*A*⋯N2^i^	0.86	2.24	3.088 (2)	167
N1—H1*B*⋯N2^ii^	0.86	2.56	3.349 (2)	153

Symmetry codes: (i) 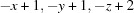; (ii) 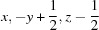.

**Fig. 1.**
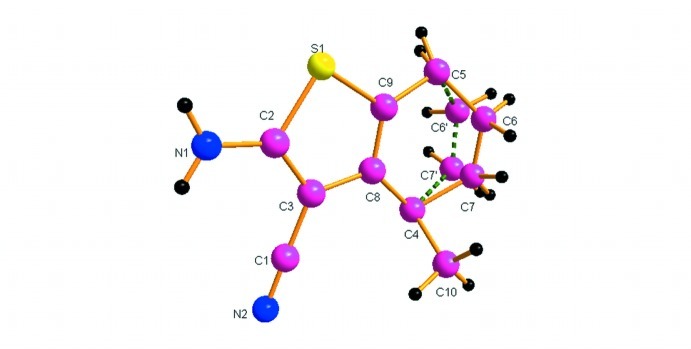
The molecular structure of the title compound with the atom numbering scheme. Displacement ellipsoids are drawn at the 30% probability level. H atoms are presented as small spheres of arbitrary radius. C6 and C7 are disordered over sites C6/C6' and C7/C7' respectively.

**Fig. 2.**
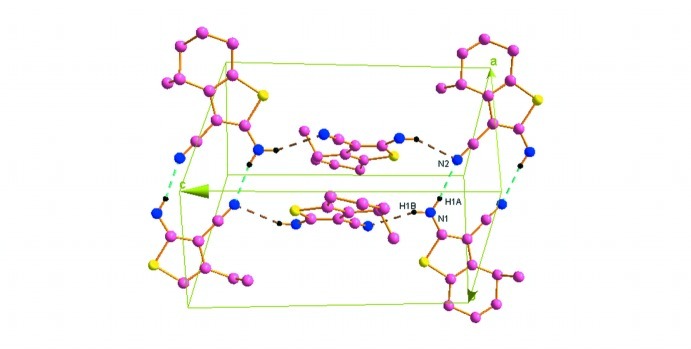
A view of the intermolecular hydrogen bonds (dotted lines) in the crystal structure of the title compound. H atoms non participating in H-bonding were omitted for clarity.

## supplementary crystallographic information

### Comment

Tetrahydro-benzothiophenes are important class of compounds which exhibits antibacterial and antifungal activities (Shetty *et al.*,2009). In the title compound, the tetrahydro-benzothiophene ring is substituted with the methyl group at C8, amine at C2 and carbonitrile group at C3 positions. The thiophene ring is essentially planar (r.m.s. deviation = 0.03 Å). The atoms C6 and C7 are disordered over two sites (C6/C6' and C7/C7') with site occupancy factors 0.810 (5) and 0.190 (5) resulting in a major and a minor conformers, respectively. The cyclohexene ring in both the conformers is in the half-chair conformation with C6 and C7 atoms being deviated from the rest of the ring atoms by 0.3330 (3) and -0.3132 (3) Å for the major conformer. The C6' and C7' atoms are deviated by -0.3738 (2) and 0.3546 (2) Å for the minor conformer respectively. The methyl group of the cyclohexene ring is oriented axially which is characterized by the bond angles C6—C8—C11 = 112.50 (2)° and C10—C8—C11 = 115.02 (2)°. The crystal structure is stabilized by two types of N—H···N intermolecular interactions generating centrosymmetric head-to-head dimers corresponding to graph-set *R*^2^_2_(12) motif (Bernstein *et al.*, 1995) and a 20-membered macrocyclic ring involving six molecules (Fig. 2). The bond distances and angles in the title compound agree very well with the corresponding bond distances and angles reported in a closely related compound (Ziaulla *et al.*, 2011).

### Experimental

To a well stirred mixture of 2-methyl cyclohexanone (8 g, 71.4 mmole) and malononitrile (4.712 g, 71.4 mmole) in ethanol (100 ml) was added elemental sulfur (2.3 g, 72 mmole). To this cooled reaction mixture was added diethyl amine (5 ml) with vigorous stirring during 1 min. The reaction mixture was stirred at 333 K for about 1 h. The solvent was evaporated under reduced pressure. The residue was poured into crushed ice and the solid obtained was purified by column chromatography (yield = 9.3 g (68%), m.p. = 392–394 K. The crystals suitable for X-ray crystallographic analysis were grown from a solution of dichloromethane.

### Refinement

The occupancies were refined individually for the C atoms C6 and C7, the disordered atoms were grouped in Part 1 and Part 2 as Part 1: C6 and C7 with partial occupancy of 0.810 (5) and Part 2: C6' and C7' with partial occupancy 0.190 (5). In this way the occupancy disordered was modelled using the EADP command in *SHELXL97*. The H atoms were placed at calculated positions in the riding model approximation with N—H = 0.86° A, C—H = 0.97 and 0.96 Å for heterocyclic and methyl H atoms respectively, with *U*_iso_(H) = 1.5*U*_eq_(C) for methyl H atoms and *U*_iso_(H) = 1.2*U*_eq_(N/C).

### Crystal data

**Table:**

C_10_H_12_N_2_S	*F*(000) = 408
*M**_r_* = 192.28	*D*_x_ = 1.271 Mg m^−^^3^
Monoclinic, *P*2_1_/*c*	Mo *K*α radiation, λ = 0.71073 Å
Hall symbol: -P 2ybc	Cell parameters from 2195 reflections
*a* = 9.6771 (2) Å	θ = 2.1–27.0°
*b* = 7.6364 (2) Å	µ = 0.28 mm^−^^1^
*c* = 13.8156 (3) Å	*T* = 296 K
β = 100.221 (2)°	Block, yellow
*V* = 1004.75 (4) Å^3^	0.18 × 0.16 × 0.16 mm
*Z* = 4	

### Data collection

**Table:**

Bruker SMART APEX CCD detector diffractometer	2195 independent reflections
Radiation source: fine-focus sealed tube	1812 reflections with *I* > 2σ(*I*)
Graphite monochromator	*R*_int_ = 0.024
ω scans	θ_max_ = 27.0°, θ_min_ = 2.1°
Absorption correction: multi-scan (*SADABS*; Bruker, 1998)	*h* = −12→12
*T*_min_ = 0.952, *T*_max_ = 0.957	*k* = −9→9
8861 measured reflections	*l* = −17→17

### Refinement

**Table:**

Refinement on *F*^2^	Primary atom site location: structure-invariant direct methods
Least-squares matrix: full	Secondary atom site location: difference Fourier map
*R*[*F*^2^ > 2σ(*F*^2^)] = 0.039	Hydrogen site location: inferred from neighbouring sites
*wR*(*F*^2^) = 0.118	H-atom parameters constrained
*S* = 1.06	*w* = 1/[σ^2^(*F*_o_^2^) + (0.0678*P*)^2^ + 0.1371*P*] where *P* = (*F*_o_^2^ + 2*F*_c_^2^)/3
2195 reflections	(Δ/σ)_max_ < 0.001
125 parameters	Δρ_max_ = 0.16 e Å^−^^3^
0 restraints	Δρ_min_ = −0.25 e Å^−^^3^

### Special details

**Table:**

Geometry. All s.u.'s (except the s.u. in the dihedral angle between two l.s. planes) are estimated using the full covariance matrix. The cell s.u.'s are taken into account individually in the estimation of s.u.'s in distances, angles and torsion angles; correlations between s.u.'s in cell parameters are only used when they are defined by crystal symmetry. An approximate (isotropic) treatment of cell s.u.'s is used for estimating s.u.'s involving l.s. planes.
Refinement. Refinement of *F*^2^ against ALL reflections. The weighted *R*-factor *wR* and goodness of fit *S* are based on *F*^2^, conventional *R*-factors *R* are based on *F*, with *F* set to zero for negative *F*^2^. The threshold expression of *F*^2^ > 2σ(*F*^2^) is used only for calculating *R*-factors(gt) *etc*. and is not relevant to the choice of reflections for refinement. *R*-factors based on *F*^2^ are statistically about twice as large as those based on *F*, and *R*- factors based on ALL data will be even larger.

### Fractional atomic coordinates and isotropic or equivalent isotropic displacement parameters (Å^2^)

**Table:**

	*x*	*y*	*z*	*U*_iso_*/*U*_eq_	Occ. (<1)
S1	0.71234 (4)	0.00203 (5)	0.83213 (3)	0.05244 (18)	
C3	0.65478 (15)	0.14987 (19)	0.98594 (10)	0.0421 (3)	
C1	0.59583 (16)	0.2640 (2)	1.04856 (10)	0.0472 (4)	
N2	0.54845 (17)	0.3532 (2)	1.10080 (11)	0.0653 (4)	
C2	0.63578 (15)	0.17225 (19)	0.88557 (10)	0.0439 (3)	
C9	0.76862 (16)	−0.0986 (2)	0.94522 (11)	0.0481 (4)	
C8	0.73216 (16)	−0.00656 (18)	1.01974 (11)	0.0420 (3)	
N1	0.57113 (16)	0.3034 (2)	0.82953 (10)	0.0650 (4)	
H1A	0.5345	0.3890	0.8566	0.078*	
H1B	0.5666	0.3014	0.7668	0.078*	
C10	0.8661 (2)	0.0583 (3)	1.18824 (14)	0.0791 (6)	
H10A	0.8837	0.0168	1.2549	0.119*	
H10B	0.8258	0.1734	1.1861	0.119*	
H10C	0.9528	0.0626	1.1637	0.119*	
C4	0.76560 (17)	−0.0641 (2)	1.12553 (12)	0.0528 (4)	
H4	0.6773	−0.0591	1.1509	0.063*	0.810 (5)
C5	0.8427 (2)	−0.2719 (2)	0.95380 (14)	0.0663 (5)	
H5A	0.9131	−0.2740	0.9118	0.080*	0.810 (5)
H5B	0.7762	−0.3655	0.9336	0.080*	0.810 (5)
C7	0.8107 (4)	−0.2568 (4)	1.1298 (2)	0.0708 (8)	0.810 (5)
H7A	0.8542	−0.2857	1.1966	0.085*	0.810 (5)
H7B	0.7281	−0.3300	1.1123	0.085*	0.810 (5)
C6	0.9126 (4)	−0.2970 (4)	1.06146 (19)	0.0730 (8)	0.810 (5)
H6A	0.9452	−0.4168	1.0715	0.088*	0.810 (5)
H6B	0.9934	−0.2202	1.0766	0.088*	0.810 (5)
C4'	0.76560 (17)	−0.0641 (2)	1.12553 (12)	0.0528 (4)	0.00
H4A	0.6823	−0.0925	1.1538	0.063*	0.190 (5)
C5'	0.8427 (2)	−0.2719 (2)	0.95380 (14)	0.0663 (5)	0.00
H5C	0.9410	−0.2557	0.9498	0.080*	0.190 (5)
H5D	0.8009	−0.3485	0.9006	0.080*	0.190 (5)
C7'	0.8760 (17)	−0.2149 (18)	1.1356 (10)	0.0708 (8)	0.190 (5)
H7C	0.8836	−0.2697	1.1996	0.085*	0.190 (5)
H7D	0.9673	−0.1679	1.1297	0.085*	0.190 (5)
C6'	0.8294 (17)	−0.3534 (17)	1.0531 (9)	0.0730 (8)	0.190 (5)
H6C	0.8883	−0.4567	1.0650	0.088*	0.190 (5)
H6D	0.7329	−0.3882	1.0528	0.088*	0.190 (5)

### Atomic displacement parameters (Å^2^)

**Table:**

	*U*^11^	*U*^22^	*U*^33^	*U*^12^	*U*^13^	*U*^23^
S1	0.0652 (3)	0.0563 (3)	0.0354 (2)	0.01326 (18)	0.00757 (18)	−0.00553 (16)
C3	0.0464 (7)	0.0441 (8)	0.0358 (7)	−0.0006 (6)	0.0074 (5)	−0.0036 (6)
C1	0.0574 (9)	0.0472 (8)	0.0362 (7)	0.0016 (7)	0.0063 (6)	−0.0003 (6)
N2	0.0859 (11)	0.0645 (9)	0.0477 (8)	0.0141 (8)	0.0176 (7)	−0.0073 (7)
C2	0.0486 (8)	0.0458 (8)	0.0369 (7)	0.0041 (6)	0.0062 (6)	−0.0016 (6)
C9	0.0532 (8)	0.0470 (9)	0.0435 (8)	0.0064 (7)	0.0073 (6)	0.0019 (6)
C8	0.0429 (7)	0.0449 (8)	0.0373 (8)	−0.0026 (6)	0.0044 (6)	0.0023 (6)
N1	0.0891 (11)	0.0662 (9)	0.0391 (7)	0.0320 (8)	0.0095 (7)	0.0061 (6)
C10	0.0743 (13)	0.1082 (17)	0.0479 (11)	−0.0107 (12)	−0.0082 (9)	0.0044 (11)
C4	0.0556 (9)	0.0618 (10)	0.0413 (8)	−0.0008 (8)	0.0095 (7)	0.0107 (8)
C5	0.0798 (12)	0.0545 (10)	0.0669 (12)	0.0193 (9)	0.0196 (9)	0.0058 (9)
C7	0.083 (2)	0.0661 (16)	0.0646 (14)	0.0052 (14)	0.0166 (15)	0.0268 (12)
C6	0.0775 (19)	0.0668 (16)	0.0730 (15)	0.0256 (14)	0.0090 (14)	0.0181 (12)
C4'	0.0556 (9)	0.0618 (10)	0.0413 (8)	−0.0008 (8)	0.0095 (7)	0.0107 (8)
C5'	0.0798 (12)	0.0545 (10)	0.0669 (12)	0.0193 (9)	0.0196 (9)	0.0058 (9)
C7'	0.083 (2)	0.0661 (16)	0.0646 (14)	0.0052 (14)	0.0166 (15)	0.0268 (12)
C6'	0.0775 (19)	0.0668 (16)	0.0730 (15)	0.0256 (14)	0.0090 (14)	0.0181 (12)

### Geometric parameters (Å, º)

**Table:**

S1—C2	1.7256 (14)	C4—C7	1.533 (3)
S1—C9	1.7397 (16)	C4—H4	0.9800
C3—C2	1.3768 (19)	C5—C6	1.533 (3)
C3—C1	1.417 (2)	C5—H5A	0.9700
C3—C8	1.443 (2)	C5—H5B	0.9700
C1—N2	1.146 (2)	C7—C6	1.513 (5)
C2—N1	1.3499 (19)	C7—H7A	0.9700
C9—C8	1.345 (2)	C7—H7B	0.9700
C9—C5	1.500 (2)	C6—H6A	0.9700
C8—C4	1.505 (2)	C6—H6B	0.9700
N1—H1A	0.8600	C7'—C6'	1.56 (2)
N1—H1B	0.8600	C7'—H7C	0.9700
C10—C4	1.507 (3)	C7'—H7D	0.9700
C10—H10A	0.9600	C6'—H6C	0.9700
C10—H10B	0.9600	C6'—H6D	0.9700
C10—H10C	0.9600		
			
C2—S1—C9	92.20 (7)	C10—C4—H4	106.5
C2—C3—C1	122.84 (14)	C7—C4—H4	106.5
C2—C3—C8	113.43 (13)	C9—C5—C6	108.02 (16)
C1—C3—C8	123.61 (13)	C9—C5—H5A	110.1
N2—C1—C3	178.40 (17)	C6—C5—H5A	110.1
N1—C2—C3	129.43 (14)	C9—C5—H5B	110.1
N1—C2—S1	120.27 (11)	C6—C5—H5B	110.1
C3—C2—S1	110.29 (11)	H5A—C5—H5B	108.4
C8—C9—C5	125.99 (15)	C6—C7—C4	112.5 (2)
C8—C9—S1	111.95 (12)	C6—C7—H7A	109.1
C5—C9—S1	122.00 (12)	C4—C7—H7A	109.1
C9—C8—C3	112.13 (14)	C6—C7—H7B	109.1
C9—C8—C4	123.39 (14)	C4—C7—H7B	109.1
C3—C8—C4	124.46 (14)	H7A—C7—H7B	107.8
C2—N1—H1A	120.0	C7—C6—C5	110.9 (3)
C2—N1—H1B	120.0	C7—C6—H6A	109.5
H1A—N1—H1B	120.0	C5—C6—H6A	109.5
C4—C10—H10A	109.5	C7—C6—H6B	109.5
C4—C10—H10B	109.5	C5—C6—H6B	109.5
H10A—C10—H10B	109.5	H6A—C6—H6B	108.1
C4—C10—H10C	109.5	C6'—C7'—H7C	109.8
H10A—C10—H10C	109.5	C6'—C7'—H7D	109.8
H10B—C10—H10C	109.5	H7C—C7'—H7D	108.3
C8—C4—C10	112.49 (15)	C7'—C6'—H6C	110.0
C8—C4—C7	109.16 (16)	C7'—C6'—H6D	110.0
C10—C4—C7	115.0 (2)	H6C—C6'—H6D	108.4
C8—C4—H4	106.5		
			
C2—C3—C1—N2	158 (6)	C2—C3—C8—C9	−0.47 (19)
C8—C3—C1—N2	−17 (6)	C1—C3—C8—C9	175.63 (14)
C1—C3—C2—N1	5.0 (3)	C2—C3—C8—C4	−178.94 (14)
C8—C3—C2—N1	−178.86 (16)	C1—C3—C8—C4	−2.8 (2)
C1—C3—C2—S1	−176.09 (12)	C9—C8—C4—C10	114.82 (19)
C8—C3—C2—S1	0.05 (16)	C3—C8—C4—C10	−66.9 (2)
C9—S1—C2—N1	179.29 (14)	C9—C8—C4—C7	−14.1 (3)
C9—S1—C2—C3	0.28 (12)	C3—C8—C4—C7	164.16 (19)
C2—S1—C9—C8	−0.56 (13)	C8—C9—C5—C6	−18.6 (3)
C2—S1—C9—C5	176.80 (15)	S1—C9—C5—C6	164.40 (18)
C5—C9—C8—C3	−176.56 (16)	C8—C4—C7—C6	44.6 (3)
S1—C9—C8—C3	0.67 (17)	C10—C4—C7—C6	−82.9 (3)
C5—C9—C8—C4	1.9 (3)	C4—C7—C6—C5	−64.8 (4)
S1—C9—C8—C4	179.16 (12)	C9—C5—C6—C7	47.9 (3)

### Hydrogen-bond geometry (Å, º)

**Table:**

*D*—H···*A*	*D*—H	H···*A*	*D*···*A*	*D*—H···*A*
N1—H1*A*···N2^i^	0.86	2.24	3.088 (2)	167
N1—H1*B*···N2^ii^	0.86	2.56	3.349 (2)	153

Symmetry codes: (i) −*x*+1, −*y*+1, −*z*+2; (ii) *x*, −*y*+1/2, *z*−1/2.
